# Changes in health workers' malaria diagnosis and treatment practices in Kenya

**DOI:** 10.1186/1475-2875-10-1

**Published:** 2011-01-07

**Authors:** Elizabeth Juma, Dejan Zurovac

**Affiliations:** 1 Ministry of Public Health & Sanitation, Division of Malaria Control, P.O. Box 19982-00202, Nairobi, Kenya; 2 Malaria Public Health and Epidemiology Group, KEMRI/Wellcome Trust Research Programme, PO Box 43640, 00100 GPO, Nairobi, Kenya; 3 Centre for Tropical Medicine, Nuffield Department of Clinical Medicine, University of Oxford, CCVTM, Oxford, UK; 4 Center for International Health and Development, Boston University School of Public Health, 85 East Concord Street, 5th Floor, Boston, MA 02118, USA

## Abstract

**Background:**

Change of Kenyan treatment policy for uncomplicated malaria from sulphadoxine-pyrimethamine to artemether-lumefantrine (AL) was accompanied by revised recommendations promoting presumptive malaria diagnosis in young children and, wherever possible, parasitological diagnosis and adherence to test results in older children and adults. Three years after the policy implementation, health workers' adherence to malaria diagnosis and treatment recommendations was evaluated.

**Methods:**

A national cross-sectional, cluster sample survey was undertaken at public health facilities. Data were collected using quality-of-care assessment methods. Analysis was restricted to facilities with AL in stock. Main outcomes were diagnosis and treatment practices for febrile outpatients stratified by age, availability of diagnostics, use of malaria diagnostic tests, and test result.

**Results:**

The analysis included 1,096 febrile patients (567 aged <5 years and 529 aged ≥5 years) at 88 facilities with malaria diagnostics, and 880 febrile patients (407 aged <5 years and 473 aged ≥5 years) at 71 facilities without malaria diagnostic capacity. At all facilities, 19.8% of young children and 28.7% of patients aged ≥5 years were tested, while at facilities with diagnostics, 33.5% and 53.7% were respectively tested in each age group. Overall, AL was prescribed for 63.6% of children aged <5 years and for 65.0% of patients aged ≥5 years, while amodiaquine or sulphadoxine-pyrimethamine monotherapies were prescribed for only 2.0% of children and 3.9% of older children and adults. In children aged <5 years, AL was prescribed for 74.7% of test positive, 40.4% of test negative and 60.7% of patients without test performed. In patients aged ≥5 years, AL was prescribed for 86.7% of test positive, 32.8% of test negative and 58.0% of patients without test performed. At least one anti-malarial treatment was prescribed for 56.6% of children and 50.4% of patients aged ≥5 years with a negative test result.

**Conclusions:**

Overall, malaria testing rates were low and, despite different age-specific recommendations, only moderate differences in testing rates between the two age groups were observed at facilities with available diagnostics. In both age groups, AL use prevailed, and prior ineffective anti-malarial treatments were nearly non-existent. The large majority of test positive patients were treated with recommended AL; however, anti-malarial treatments for test negative patients were widespread, with AL being the dominant choice. Recent change of diagnostic policy to universal testing in Kenya is an opportunity to improve upon the quality of malaria case management. This will be, however, dependent upon the delivery of a comprehensive case management package including large scale deployment of diagnostics, good quality of training, post-training follow-up, structured supervisory visits, and more intense monitoring.

## Background

Health workers' adherence to diagnostic and treatment guidelines is one of the critical aspects determining effective implementation of malaria case management policies [1]. In line with international recommendations [2], in 2004 the first-line treatment policy for uncomplicated malaria in Kenya was changed from ineffective sulphadoxine-pyrimethamine (SP) [3] to artemisinin-based combination therapy (ACT). A specific ACT - artemether-lumefantrine (AL) - was recommended for patients weighing 5 kg and above, quinine was recommended for children below 5 kg and pregnant women, SP was reserved for intermittent preventive treatment in pregnancy (IPTp), and amodiaquine (AQ), a prior and failing second-line option [4], was no longer recommended for malaria treatment. To support the change of treatment policy, malaria diagnostic policies were also revised to promote presumptive treatment of fever in children under five years of age and, wherever available, microscopy or rapid diagnostic tests (RDT) were recommended for testing febrile patients aged five years and older, with subsequent treatment of only test positive cases with AL [5].

By the end of 2006, the new treatment policy was implemented countrywide [6]. Regarding malaria diagnostics, health workers were trained on the use of RDTs during the in-service case management training; however, only limited quantities of RDTs were supplied, and most of the malaria diagnostic capacities in the country relied on the availability of malaria microscopy [7]. The findings from several studies [8-10] evaluating health workers' adherence to age-specific guidelines during the early AL implementation phase have revealed that: 1) overall AL use was low in both age groups, with prevailing practice of prescribing non-recommended AQ, SP and their combinations; 2) parasitological diagnosis was underused in older children and adults while at variance with diagnostic recommendations, a substantial proportion of young children was tested; 3) prescriptions of AL largely followed test results and recommendations - however, most test negative patients were still treated for malaria, mainly with alternative and non-recommended treatments.

Between 2007 and 2009, the Kenyan Division of Malaria Control reinforced health workers' case management practices to adhere to case management guidelines. In this paper, malaria diagnosis and treatment practices observed during the national malaria case management survey undertaken in 2010 are reported, approximately three years after the new AL policy was implemented countrywide.

## Methods

### Survey design and data collection

A cross-sectional, cluster sample, health facility survey was undertaken in public facilities between 18^th ^January and 12^th ^February 2010. From the universe of 6,094 public facilities in Kenya the following categories of facilities were excluded from the sampling frame: 1) facilities from Nairobi province due to absence of malaria transmission and requiring special studies to evaluate malaria case management, 2) tertiary hospitals since they serve mainly as referral facilities, and 3) facilities run by other than Ministry of Health (MoH) and Local Authorities (LA) because they provide services to special patient groups such as military or prisoners. In total, 861 facilities were excluded and therefore our sampling frame consisted of 5,233 health facilities. For the purpose of sampling, facilities belonging to the faith-based and non-governmental organizations were classified into one category. Similarly, MOH and LA owned facilities were grouped into government category as well as smaller facilities such as dispensaries and health centres which also represented one category. Therefore, in each of seven provinces, four strata based on the facility type (hospitals versus smaller facilities) and ownership (government versus faith based/non-government) were formed. Finally, from each of the 28 strata, a simple, random sample proportional to the number of facilities in a stratum was drawn. A cluster was defined as all outpatient encounters between health workers and patients occurring on a survey day.

Data at each facility were collected over a single survey day using a range of quality-of-care methods including health facility assessments, health worker interviews, and exit interviews with caretakers and patients. Firstly, all patients presenting to the outpatient departments underwent rapid screening when they were ready to leave the facility. Non-referred and non-pregnant patients weighing 5 kg and above and presenting for an initial outpatient visit with fever underwent a detailed interview. Of relevance for this report, the information about patients' age, weight, temperature, main complaints, routine malaria diagnostics requested, results reported and medications prescribed was collected during the exit interview and from the patient-held cards. Secondly, each facility was assessed to determine the survey day availability of anti-malarial drugs, antibiotics, malaria RDTs and functional malaria microscopy service. Finally, at the end of the working day, all health workers who saw recruited patients on the survey day were interviewed to collect information on their demographics and exposure to malaria case management interventions. Informed written consent was obtained for all participants.

### Definitions and analytical approaches

The study definitions reflected national guidelines for management of uncomplicated malaria, which were valid at the time of the study [11]. In summary, the guidelines recommend that in high malaria risk areas, all children under five years of age with fever or history of fever should be presumptively treated with AL. In low malaria risk areas, presumptive treatment with AL is recommended in all febrile children in the absence of measles, runny nose and other obvious causes of fever. For clinical management purposes, all parts of Kenya were classified as high malaria risk areas, except the highlands of Central and Nairobi provinces. In patients aged five years and older and regardless of the malaria risk, all febrile patients in the absence of another obvious cause of fever should be tested for malaria (microscopy or RDT) and only test positive patients should be treated with AL. In the same age group, presumptive AL treatment is recommended in absence of malaria diagnostics.

Therefore, to reflect criteria for testing and AL treatment, all analyses were restricted to febrile, non-pregnant patients weighing 5 kg and above, presenting for an initial outpatient visit without being referred or admitted for hospitalization. Patients aged five years and older presenting with another obvious cause of fever, as well as the children less than five years of age meeting the same criteria in low risk areas, were excluded from the analysis. Another obvious cause of fever was defined as febrile patients presenting concomitantly with runny nose, sore throat, oral thrush, wounds, urinary problem, skin problem or abscesses. Fever was defined as axillary temperature of ≥37.5°C or a history of fever during the present illness.

To ensure comparable evaluation of anti-malarial treatment practices based on different age-specific recommendations for malaria diagnosis, the focus of the analysis was observations from health facilities where AL was in stock during the survey, stratified by the availability of diagnostics and patients' age (under five and over five years of age). At facilities with malaria diagnostics, anti-malarial treatment practices were further stratified by the use of malaria diagnostic tests and the patient's test result. Combined microscopy and RDT results are presented since the small number of patients with malaria RDTs precluded a meaningful analysis stratified by type of diagnostics.

Data entry and management was undertaken using Access (Microsoft, USA), through customized data entry screens with in-built range and consistency checks. All forms were entered twice by independent data entry clerks and data files were compared for errors using a verification programme and referring to original questionnaires. All analyses were performed using STATA, version 11. The precision of proportions (95% confidence interval [CI]) was determined adjusting for the cluster sampling at the health facility level.

### Ethical approval

Ethical approval for the survey was provided by the Kenyatta National Hospital/University of Nairobi-Ethics & Research Committee (reference number KNH-ERC/A/383).

## Results

### Sample description

The survey was undertaken at 174 public health facilities, of which the majority (70.1%) were government facilities, followed by faith-based (25.9%) and non-governmental organization facilities (1.2%). Most facilities were dispensaries (70.1%), while health centres and hospitals accounted for 18.4% and 11.5% of facilities, respectively. Parasitological diagnosis of malaria was available on the survey day at 96 (55.2%) facilities, more commonly using malaria microscopy (88/96; 91.7%) than RDTs (13/96; 13.5%). Five health facilities had both diagnostic capacities. AL was available in 94.3% facilities, while SP tablets, quinine injections and quinine tablets were respectively in stock at 88.5%, 77.6% and 69.0% of facilities. Non-recommended AQ was available in only 23.6% of facilities.

Malaria diagnosis and treatment practices were analysed for 1,976 febrile patients who met the inclusion criteria (974 aged <5 years and 1,002 aged ≥5 years), at 159 facilities where AL was in stock on the survey day. At five facilities with AL in stock, no febrile patients meeting the inclusion criteria were seen during the survey days. Of these 1,976 febrile patients, 1,096 (567 aged <5 years and 529 aged ≥5 years) were seen at 88 facilities with malaria diagnostic support. The remaining 880 patients (407 aged <5 years and 473 aged ≥5 years) were seen at 71 facilities without malaria diagnostic capacity.

### Malaria testing and routine test positivity rates

Table 1 presents age-specific malaria testing rates for 1,976 febrile patients seen at all study facilities and 1,096 patients seen at facilities with malaria diagnostic support. At all facilities, 24.3% (95% CI: 19.0-29.7) of febrile patients were tested. At facilities with malaria diagnostic support, 43.2% (95% CI: 36.4-50.1) were tested. Among patients aged ≥5 years, 53.7% (95% CI: 45.4-61.9) of older children and adults were tested at facilities with diagnostic support. Interestingly, at the same facilities, 33.5% (95% CI: 25.1-42.0) of children <5 years of age had also undergone parasitological testing. Among tested patients, the routine malaria test positivity rate was 54.0% (95% CI: 45.4-62.6), with the proportion higher, although not statistically significant, in older children and adults (58.1%; 95%: 46.9-69.3) than in children <5 years of age (47.9%; 95% CI: 38.3-57.5).

**Table 1:** Malaria testing rates for febrile patients under and over 5 years of age

All health facilities	Aged < 5 years (N = 974)		Aged ≥ 5 years (N = 1,002)		All age groups (N = 1,976)	
	
	n (%)	95% CI	n (%)	95% CI	n (%)	95% CI
Tested for malaria	193 (19.8)	14.3-25.3	288 (28.7)	21.8-35.7	481 (24.3)	19.0-29.7

**Health facilities with malaria diagnostics**	**< 5 years** **(N = 567)**		**≥ 5 years** **(N = 529)**		**All age groups** **(N = 1,096)**	
	
	**n (%)**	**95% CI**	**n (%)**	**95% CI**	**n (%)**	**95% CI**

Tested for malaria	190 (33.5)	25.1-42.0	284 (53.7)	45.4-61.9	474 (43.2)	36.4-50.1

### Anti-malarial treatment practices for febrile children under five years of age

Table 2 presents anti-malarial treatment practices for febrile children <5 years of age stratified by the availability of diagnostics, use and result of malaria test. Overall, the large majority of febrile children were treated with recommended AL (63.6%; 95% CI: 58.5-68.6); 9% (95% CI: 5.8-12.1) were treated with non-recommended quinine or combination of AL and quinine (AL+QN); only 2% (95% CI: 0.5-3.6) received ineffective AQ or SP monotherapies; 25.4% (95% CI: 20.9-29.8) were not treated for malaria.

**Table 2:** Anti-malarial treatment practices for febrile children under 5 years of age stratified by the availability of diagnostics, use and result of malaria test

Children aged < 5 years	Health facilities with diagnostics			Health facilities without diagnostics	All health facilities
	
	Positive test N = 91 (%)	Negative test N = 99 (%)	Test not done N = 377 (%)	All children N = 407 (%)	Total N = 974 (%)
**AL**	68 (74.7)	40 (40.4)	229 (60.7)	282 (69.3)	619 (63.6)

**AL+QN**	17 (18.7)	6 (6.1)	22 (5.8)	16 (4.0)	61 (6.3)*

**QN**	5 (5.5)	3 (3.0)	10 (2.7)	8 (2.0)	26 (2.7)

**SP**	0	5 (5.1)	8 (2.1)	1 (0.3)	14 (1.4)

**AQ**	0	2 (2.0)	4 (1.1)	0	6 (0.6)

**QN+SP**	1 (1.1)	0	0	0	1 (0.1)

**No AM prescribed**	0	43 (43.4)	104 (27.6)	100 (24.6)	247 (25.4)

**Any AM prescribed**	91 (100)	56 (56.6)	273 (72.4)	307 (75.4)	727 (74.6)

**Antibiotic prescribed**	70 (76.9)	89 (89.9)	281 (74.5)	329 (80.8)	769 (79.0)

*All QN treatments as part of AL+QN therapy include administration of injectable quinine

AL, artemether-lumefantrine; AQ, amodiaquine; SP, sulphadoxine-pyrimethamine; QN, quinine; AM, anti-malarial.

Among test positive children, anti-malarial treatment was prescribed universally and 74.7% (95% CI: 61.0-88.5) of children were treated with AL, 18.7% (95% CI: 6.5-30.9) with AL+QN and 5.5% (95% CI: 0.8-10.2) with quinine alone. Notably, none of the test positive children were treated with ineffective AQ or SP monotherapies (Table 2). Interestingly, among test negative children, AL was prescribed for 40.4% (95% CI: 26.6-54.3) of these children, 9.1% (95% CI: 2.5-15.7) were treated with AL+QN or quinine alone and 7.1% (95% CI: 0-15.4) were treated with either AQ or SP monotherapies. At least one anti-malarial drug was prescribed for 56.6% (95% CI: 43.1-70.0) of the test negative children. AL treatment also prevailed among children without test performed at facilities with (60.7%; 95% CI: 51.2-70.3) and without (69.3%; 95% CI: 62.1-76.5) diagnostic support. Finally, across all patient categories, over 75% of children were treated with antibiotics (Table 2).

### Anti-malarial treatment practices for febrile patients aged five years and older

Table 3 presents anti-malarial treatment practices for febrile patients aged five years and older stratified by the availability of diagnostics, use and result of malaria test. Across all patient categories, the patterns of anti-malarial treatments observed in this age group were similar to those observed in children under five years of age. In summary, nearly two thirds of all patients were treated with AL (65.0%; 95% CI: 59.8-70.1), treatments with ineffective AQ or SP monotherapies were very rare (3.9%; 95% CI: 1.7-6.1), at least one anti-malarial drug was prescribed for half of the test negative patients (50.4%; 95% CI: 38.6-62.3), AL was treatment of choice for test positive patients (86.7%; 95% CI: 79.8-93.5) but also commonly used for patients with negative test (32.8%; 95% CI: 20.6-44.9), and those without test performed at facilities with (58.0%; 95% CI: 46.6-69.3) and without (69.1%; 95% CI: 61.7-76.5) diagnostic support. 68.9% (95% CI: 64.8-73.0) of patients were treated with antibiotics, yet at variance to children under five years of age, the non-recommended AL+QN treatment was less common for test positive patients (7.3%; 95% CI: 2.1-12.4).

**Table 3:** Anti-malarial treatment practices for febrile patients 5 years and older stratified by the availability of diagnostics, use and result of malaria test

**Patients aged **≥ **5 years**	Health facilities with diagnostics			Health facilities without diagnostics	All health facilities
	
	Positive test N = 165 (%)	Negative test N = 119 (%)	Test not done N = 245 (%)	All patients N = 473 (%)	Total N = 1,002 (%)
**AL**	143 (86.7)	39 (32.8)	142 (58.0)	327 (69.1)	651 (65.0)

**SP**	1 (0.6)	16 (13.5)	9 (3.7)	12 (2.5)	38 (3.8)

**AL+QN**	12 (7.3)	2 (1.7)	0	16 (3.4)	30 (3.0)*

**QN**	6 (3.6)	2 (1.7)	1 (0.4)	7 (1.5)	16 (1.6)

**AQ**	0	1 (0.8)	0	0	1 (0.1)

**Other AM**	1 (0.6)	0	1 (0.4)	2 (0.4)	4 (0.4) ^†^

**No AM prescribed**	2 (1.2)	59 (49.6)	92 (37.6)	109 (23.0)	262 (26.2)

**Any AM prescribed**	163 (98.8)	60 (50.4)	153 (62.5)	364 (77.0)	740 (73.9)

**Antibiotic prescribed**	90 (54.6)	92 (82.4)	169 (69.0)	333 (70.4)	690 (68.9)

*All QN treatments include administration of injectable quinine

^† ^Other anti-malarial treatment included dihydroartemisinin (2), QN+SP (1) and AL+SP (1).

AL, artemether-lumefantrine; AQ, amodiaquine; SP, sulphadoxine-pyrimethamine; QN, quinine; AM, anti-malarial.

## Discussion

### Adherence to testing recommendations

Between 2006 and 2009, Kenya promoted malaria diagnostic policy recommending parasitological testing for febrile older children and adults and presumptive diagnosis in young children. The findings from this 2010 study revealed low testing rates without substantial difference between children under five years of age (20%) and older children and adults (29%). While the absence of malaria diagnostics in nearly half of the facilities due to partial deployment of RDTs provides an explanation for low testing rates in older children and adults, the testing practices observed at facilities with available diagnostics are worth noting. At these facilities, despite the increase in testing rates compared with all facilities, the testing rates for febrile older children and adults did not exceed 54%, and at variance with presumptive recommendations for children under 5 years of age, 34% of these children were still tested. The testing patterns found in this study concur with reports from studies undertaken during the early AL implementation in Kenya, which suggested underuse of testing in older children and adults and important presence of testing in children under five years of age [9].

The in-service malaria case management training for health workers - the key implementation activity reinforcing age-specific recommendations for malaria testing between 2007 and 2009 - deserves special attention. The issues related to the quality of in-service training and unclear or incorrect case management messages communicated to health workers during the early AL implementation phase in 2006 were previously reported [12]. Despite the standardization of the training between 2007 and 2009 through the development of the unique curriculum [13], it should be acknowledged that the large scale, multi-cascade training programme covering more than 20,000 health workers in over 500 training sessions and implemented by 15 different organizations is unlikely to be sufficient to guarantee uniformed quality of training and to result in changes of practices if it is not supported with post-training follow-up and structured supportive supervision. Unfortunately, the last components were the weakest parts of the case management activities, which were rarely present throughout the process of the policy implementation.

Nevertheless, in 2010, Kenya has changed diagnostic policy and case management guidelines to unambiguously recommend parasitological testing of all febrile patients regardless of the age category [14]. Therefore, more positively, non-adherent practices in young children as suggested in this study can be viewed as a positive starting point under the new policy. Currently, at facilities with available diagnostics, only 43% of all febrile patients are tested, yet when compared with results reported in similar studies, this is more common than the 40% of patients tested in Uganda [15], 31% in Angola [16], 27% in Zambia [17] and 27% in Tanzania [18]. After the policy and guidelines recommendations have abandoned presumptive diagnosis in young children in Kenya, it is believed that future implementation of a comprehensive case management package based on large scale deployment of RDTs with emphasis on post-training follow up and structured supervisory activities would further increase health workers adherence to new testing recommendations.

### Change in treatment practices from ineffective anti-malarial therapies to AL

The findings of this analysis, three years after AL was delivered to health facilities in Kenya, have shown that AL treatment practices have prevailed over other anti-malarial treatments in both age groups (64% in young children and 65% in older children and adults). Despite non-adherent practices that are still present, it is important to note that the use of ineffective monotherapies such as SP or AQ was nearly non-existent. However, the use of the non-recommended combination of AL and quinine was particularly common among test positive children under five years of age (18%). These findings reveal a major shift in treatment practices compared with reports during the early AL implementation, which were characterized by low AL use in both age groups, with prevailing practice of prescribing non-recommended AQ, SP and their combinations [8-10].

There are several possible explanations for these patterns. Firstly, despite the change of policies and deployment of AL to peripheral facilities, the discontinuation of old treatment practices and the translation of new policies into recommended clinical practice is a relatively slow process. The slow changes in treatment practices observed during the early and late implementation process in Zambia [19] concur with this view. Secondly, declining availability of non-recommended AQ at Kenyan facilities is likely to be a further determinant preventing health workers' selection of ineffective treatments. In prior studies the similar findings were suggested with respect to the availability of chloroquine in Uganda under the ACT policy [20] and indeed in Kenya during the prior SP policy [21]. Thirdly, incorrect training messages on adequate AQ efficacy and its potential role in case management that were initially delivered to health workers during the early implementation phase [12] were corrected over time. Finally, with respect to the emergence of AL+QN use in test positive patients, the prior studies in Kenya have frequently shown that patients presenting with conditions that are more likely to be malaria are more commonly treated with second-line treatments or drugs perceived by health workers to be a "stronger" cure for malaria [12,22]. Furthermore, since this pattern is prominent in young children where vomiting complaints are common, the use of intramuscular quinine in combination with AL might be explained as the health worker's treatment strategy for reducing the risk of the patient's non-adherence. Yet this is the practice that should be discouraged, and among patients with non-severe febrile disease, emphasis should be put on oral AL treatments with the first dose administered at the facility under direct observation.

### Challenges in adherence to test negative results

Anti-malarial treatment practices for test negative patients deserve special attention since failure to adhere to test negative results severely compromises the cost-benefit of testing-based malaria case management strategies [23]. These findings revealed that as high as 58% of test negative young children and 50% of older children and adults are treated for malaria. In both age groups, the dominant treatment for test negative patients was AL. The prior reports from Kenya during the early implementation phase suggested that AL prescriptions largely followed test results [9] while alternative anti-malarial treatments were reserved for test negative patients. In this study, it was observed that the initial encouraging observations of most AL treatments adhering to the test negative results were reversed three years later when the overwhelming majority of treated test negative patients were indeed treated with AL.

There are several possible explanations for these patterns in this study. While in young children, the disregard of test negative patients could be explained by the promotion of presumptive treatment (but without testing) during the study period, the magnitude of practices in discordance with implemented guidelines is worrying in older children and adults. Reduced availability of non-recommended AQ in public health facilities has not resulted in more rational use of anti-malarial drugs for test negative patients but alongside more established AL policy in switch of practices from AQ to AL. However, non-adherence to test-negative results is not unique to Kenya, and larger scale evaluations have often reported similar results across Africa under the microscopy-based [18,24,25] and RDT-based [16,17,26] diagnostic strategies. Conversely, smaller scale studies undertaken under more controlled conditions have suggested that more intensive interventions, including integrated in-service training supported with supervision and strengthened monitoring and surveillance, may improve adherence to test results [27-30]. An important component to support health workers in the management of test negative patients is development and implementation of guidelines for management of non-malaria febrile illness. This component has not been addressed in 2010 in Kenya, and the opportunity lies in the forthcoming large-scale implementation of RDTs. Finally, it was observed that respectively 90% and 82% of test negative young children and patients five years and older were treated with antibiotics. More pragmatically, if prescriptions of medications are perceived as good quality of care, then the withdrawal of anti-malarial treatment for test negative patients would not substantially interfere with this perception. In most cases, the main difference would be only an omission of anti-malarial treatment from the existing polypharmacy prescriptions. Further qualitative research is required to better understand these practices.

## Conclusions

Overall, malaria testing rates were low and despite different recommendations for malaria diagnosis for patients under and over five years of age, only moderate differences in testing rates between the two age groups were observed at facilities where diagnostics were available. More positively, in both age groups, AL treatment practices have prevailed over other anti-malarial treatments and the use of ineffective monotherapies was nearly non-existent. Furthermore, the large majority of test positive patients were treated with recommended AL, while in the smaller subset of non-adherent treatments the use of AL+QN was observed. However, the use of anti-malarials for test negative patients was widespread, with AL being the dominant treatment choice. Further improvements in malaria case management are urgently required and they will be dependent upon the delivery of a comprehensive case management package including deployment of RDTs, good quality training, post-training follow-up, structured supervisory visits, and more intense monitoring. The recent change of diagnostic policy abandoning presumptive treatment and recommending universal parasitological diagnosis across all age groups should be an opportunity to strengthen the quality of malaria case management.

## Competing interests

The authors declare that they have no competing interests.

## Authors' contributions

Both authors contributed to the study design, data analysis, interpretation of the results, policy implications of the findings and drafting and finalization of the manuscript.
